# Symmetry Breaking on Density in Escaping Ants: Experiment and Alarm Pheromone Model

**DOI:** 10.1371/journal.pone.0114517

**Published:** 2014-12-31

**Authors:** Geng Li, Di Huan, Bertrand Roehner, Yijuan Xu, Ling Zeng, Zengru Di, Zhangang Han

**Affiliations:** 1 School of Systems Science, Beijing Normal University, Beijing, China; 2 Institute for Theoretical and High Energy Physics, University of Paris 6 (Pierre and Marie Curie), F-75005, Paris, France; 3 The College of Natural Resources and Environment of South China Agricultural University, Guangzhou, China; 4 School of Systems Science, Beijing Normal University, Beijing, China; Tel Aviv University, Israel

## Abstract

The symmetry breaking observed in nature is fascinating. This symmetry breaking is observed in both human crowds and ant colonies. In such cases, when escaping from a closed space with two symmetrically located exits, one exit is used more often than the other. Group size and density have been reported as having no significant impact on symmetry breaking, and the alignment rule has been used to model symmetry breaking. Density usually plays important roles in collective behavior. However, density is not well-studied in symmetry breaking, which forms the major basis of this paper. The experiment described in this paper on an ant colony displays an increase then decrease of symmetry breaking versus ant density. This result suggests that a Vicsek-like model with an alignment rule may not be the correct model for escaping ants. Based on biological facts that ants use pheromones to communicate, rather than seeing how other individuals move, we propose a simple yet effective alarm pheromone model. The model results agree well with the experimental outcomes. As a measure, this paper redefines symmetry breaking as the collective asymmetry by deducing the random fluctuations. This research indicates that ants deposit and respond to the alarm pheromone, and the accumulation of this biased information sharing leads to symmetry breaking, which suggests true fundamental rules of collective escape behavior in ants.

## Introduction

The collective behavior of large groups of animals is a truly fascinating natural phenomenon. Especially when under pressure, such as encountering predators, certain animals form groups and produce amazing patterns for survival [1], [2]. Apart from collective motion in fish schools [3]–[5] and bird flocks [6]–[9] in open space, an interesting panic-induced collective behavior is symmetry breaking, which was introduced by Helbing et al. (2000), and occurs when a crowd pedestrians escape from a closed space with two symmetrically located exits, but one of the exits is more used than the other [10].

Altshuler et al. (2005) found that symmetry breaking can even occur with escaping ants [11]. After introducing a dose of insect-repelling liquid, the resulting high panic condition induces symmetry breaking in escaping ants from a room with two symmetrically located exits. Inspired by Helbing et al. (2000) [10], they constructed a Vicsek-like model in which the velocity of ant depends not only on the velocity of itself but also on the average velocity of its neighborhoods [12]. They also investigated how the total number of ants influences symmetry breaking. Their results indicate that while the model suggests a discrete increase of the asymmetry as the number of ant increases, the experiments reveal no measurable dependence on the number of ants [11].

This conclusion that the asymmetry is independent of the density contradicts the facts reported in the literature that density is an important factor affecting the properties of collective behavior in real biological groups, such as non-living systems [13], macromolecules [14], bacterial colonies and cells [15], [16], insects [17], [18], fish schools [4], [5], as well as in self-propelled particles (SPP) models [12]. Thus, it is worthwhile to investigate the interactions inducing asymmetry in ant groups related to density. In this current research, we perform an experiment to explore whether and how symmetry breaking is dependent on the density of ants.

Although Vicsek-like models are successful in a wide range of collective motion systems, recent experimental studies uncovered that the collective behavior of specific species may not satisfy all the assumptions and rules. Some examples follow. Rather than incorporating homogeneous agents, a well-defined hierarchy has been found in pigeon flocks [8]. Different from the assumption that the interaction depends on a fixed metric distance, the starlings in flocks base their interactions on the topological distance (each bird interacts on average with six to seven nearest neighbors) [7]. In addition, some species of fishes tend to follow just one nearest neighbor [19].

In the case of ants, it is well-known that most species have poor eyesight, and a portion of ants are nearly blind. Rather than seeing how other individuals move nearby, ants use their antenna to sense their surroundings and use pheromones to communicate with each other [20]. Specifically, when under danger, alarm pheromones are used and induce specific behavior responses [21]–[23]. Based on the biological facts, we argue that the Vicsek-like model may be unsuitable for describing the ant collective behavior, and we need to construct a model incorporating alarm pheromones to elucidate the underlying rules regulating the panic escape collective behavior of ants.

## Results

### Experiment

We introduced a group of ants (*Solenopsis invicta* Buren) into a cell with two symmetrically located exits, which were initially blocked so that the ants could not escape. Then a dose of citronella was injected into the center of the cell, and the exits were opened synchronously so that the ants were able to escape. There were 291 repetitions in total. To measure the symmetry breaking, Altshuler et al. (2005) [11] used *the percentage difference in door use* (abbreviated as *difference*), which is calculated as
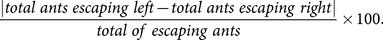
(1)


**Figure 1:**
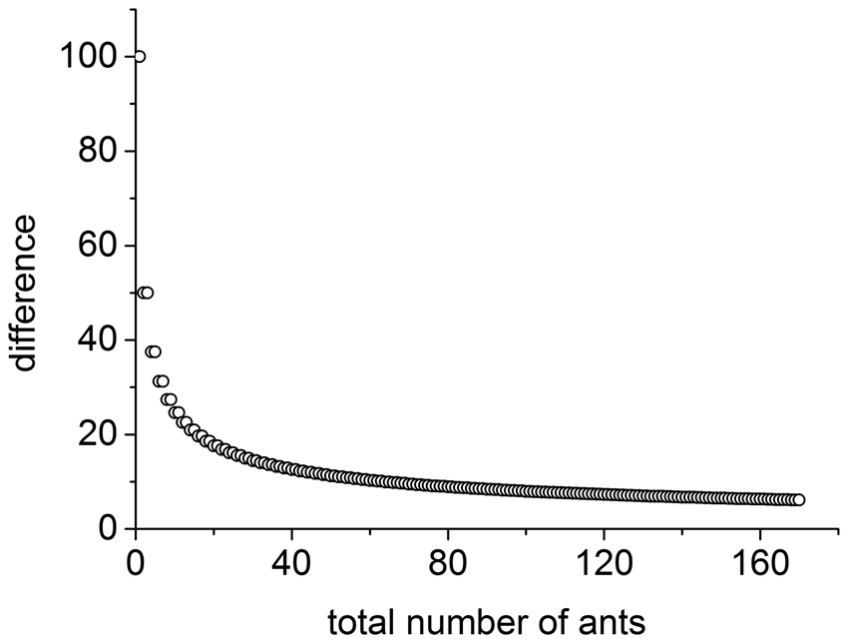
Random difference produced from random SPP model with respect to the total number of particles.

**Figure 2:**
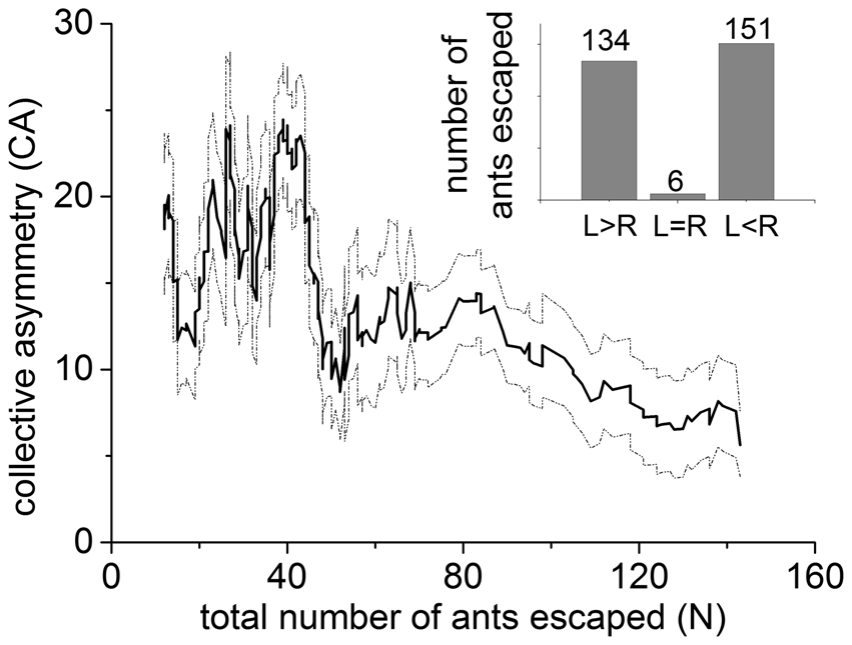
Moving average of the collective asymmetry (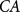) vs. the total number of escaped ants (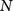). The two dashed lines above and below the curve are the 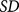
s (the standard error of the mean) of the moving average. This curve displays a high profile when the number of ants is small as well as a decrease of 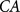 to a low profile when the number of ants is large. The inset shows, within the 291 individual experiments, that the number of experiments in which 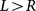, 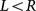 and 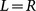 is 151, 134 and 6, respectively. 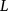 and 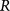 denotes the total number of ants escaping left and right, respectively. This indicates that there are no hidden biased environmental factors affecting the direction of ant movement.

This measure, however, does not take into account the natural random difference introduced by a random SPP model. The basic idea is that randomness leads to symmetry, whereas the symmetry breaking is observed when induced by collective behavior other than randomness. The difference, however, has a non-zero value even in a random SPP escape. Taking a two-particle SPP escape as an example, the total number of ants escaping left and right can be (2, 0), (1, 1) and (0, 2). The difference is 50% and not zero. The focus of this current research is to study how the symmetry breaking is influenced by the total number of ants, which is actually the density when the experiment cell is fixed. Thus, taking two experiments with different total ant numbers, 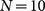 and 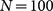, as another example, the 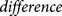 for a random SPP escape is 24.6 and 8.0, respectively, as demonstrated in Fig. 1. By subtracting the randomness-induced part from the 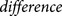, we introduce a new measurement to measure the symmetry breaking, collective asymmetry (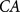), which is given by
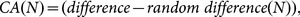
(2)where 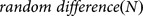 denotes the average of random difference produced from an 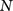 particle random SPP model (see *Materials and Methods*). The total number of ants escaped cannot be controlled precisely in each experiment because the number of ants introduced into the cell is imprecise and not all the ants are able to escape (see *Materials and Methods*). Therefore, it is impossible to test symmetry breaking by repeating the experiment to obtain the average of 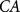 with a fixed specific total ant number. Therefore, a moving average of 31 points is calculated for the 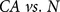 curve as illustrated in Fig. 2. The two dashed lines above and below the curve are the 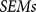 (the standard error of the mean) of the 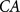. The period for the moving average is discussed in the discussion to detail the robustness of the 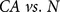 relationship. The results show that there is a nonlinear relationship between the degree of symmetry breaking and the total number of escaped ants. This curve has a high profile when the number of ants is small as well as a decrease of 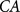 to a low profile as the number of ants grows higher (Fig. 2). The number of experiments in which 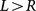, 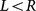 and 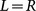 is 151, 134 and 6, respectively, as shown in the inset of Fig. 2. 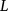 and 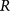 denotes the total number of ants escaping left and right, respectively. This means that there are no hidden biased environmental factors affecting the direction of ant movement. We will demonstrate the discrepancy between the results of a Vicsek-like model with the alignment rule and our presented experimental results. Then, we will set up an alarm pheromone model based on ants biological features and demonstrate its greater explanatory power.

### Simulation of Vicsek-like Model

**Figure 3:**
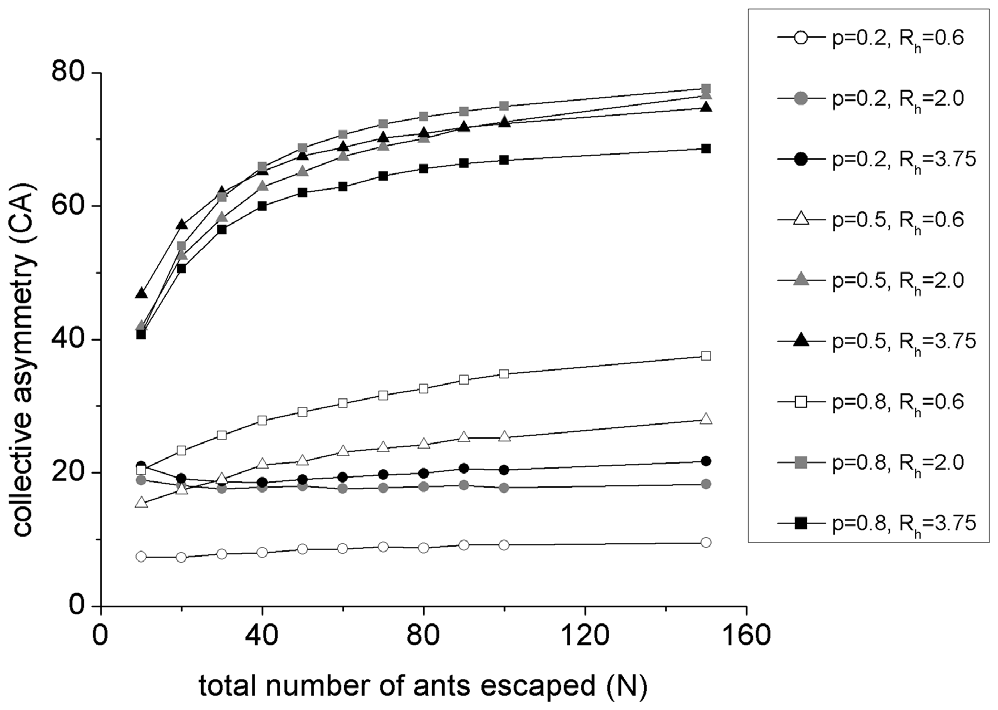
Percentage of 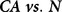 of Vicsek-like model. 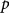
 is assigned a value of 0.2 (circle or dots), 0.5 (triangle) and 0.8 (square). 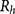 is assigned a value of 0.6 (hollow), 2.0 (gray) and 3.75 (black). The black square (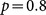, 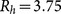) corresponds to parameter values as in Altshuler's model (11). All the points are averaged over 10000 runs of simulation.

The Vicsek-like model used by Altshuler et al. inspired by Helbing et al. (2000) suggests a discrete increase of the 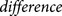 as the number of ant increases [11]. As analyzed above, for small total number of ants, the random difference is large and for a large total number it is small. Thus, an increase of 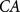 as the total number of ants increases is suggested by Altshuler's simulation result, which does not match our experimental outcome. We interpret the core mechanism for the Altshuler's model as the alignment rule proposed in the Vicsek model. A simpler model is established which contains the alignment rule and being reflected by the wall, eliminating the bouncing interaction among ants and the interaction between ants and the central repellent spot. A total number of 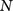 virtual ants are introduced into a circular cell with two symmetric exits. The initial positions and the directions of movement of ants are chosen randomly, and they move with a constant absolute velocity 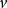. If an ant gets closer to one of the two exits than a certain critical radius, 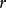, it escapes through the door. When an ant hits the wall of the cell, it is reflected. Otherwise, the unit vector of velocity of an ant is calculated as follows:
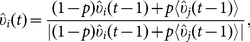
(3)where 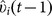 is the unit vector of the ants velocity at computer time step 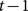, and 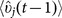 is the average unit vector of the velocities of neighboring ants within a radius 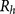 from the ant under study at computer step 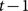. 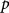 is a parameter that determines the tendency of an ant to align with its neighbors. The main parameters influencing the phenomenon of symmetry breaking are 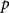 and 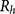. For example, when 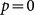 or 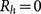, the ants will behave as random particles and induce low asymmetry. 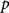 is assigned values of 0.2, 0.5 and 0.8. 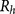 is assigned values of 0.6, 2.0 and 3.75. This produces 9 parameter combinations. The radius of the cell 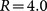 and 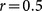, which is the same as that both in Altshuler's model and in our experiment. The velocity is set as 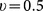 cm per time step, which corresponds to the expected value used in Altshuler's model without considering the velocity distribution. The simulation results suggest an increase of the 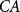 as the number of ants increases as is shown in Fig. 3. This simpler model produces the same qualitative results as the Altshuler's model, and both models cannot explain the experimental results, which indicate a significant decline of 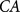 when 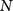 is large enough. This suggests that the alignment rule is the core mechanism in these two models, and it is not suitable for describing the underlying rule of the panic-induced collective behavior of ants.

### Simulation of Alarm pheromone model

A number of pheromone-related models, including the best known ant colony algorithms, have been developed [24]. However, almost all of them are not situated to describe the panic-induced collective behavior of ants. On one hand, most of the models are delicately constructed for solving optimization problems rather than exploring biological laws in nature. On the other hand, nearly all of them are directly inspired by foraging behavior induced by recruitment pheromones, which is different from the alarm pheromone-triggered behavior in this current case. For example, the ants do not have to move between a source and a sink. Effective communication of alarm can be critical for social animals thus they are able to address threats posed by predators and competitors. Decades ago, Wilson defined alarm behavior in fire ants as the rapid, erratic movement of workers toward a disturbed worker [25]. The behavioral responses of large or better defended societies (i.e., red imported fire ants, which were used in our experiment) to alarm are basically the same. They are attracted toward the source at low pheromone concentration and at high concentration go into frenzied activity, occasionally attacking the pheromone source [23], [26]. Inspired by these biological studies, we assume that when moving under panic, an ant deposits a constant amount of alarm pheromone that evaporates after being released and has a tendency to orient to the position where the concentration of alarm pheromone is maximum within its detection range. Moreover, taking into account the limitations of the biological perception, we suppose that if the detected amounts of pheromone in different lattices are larger than a threshold, an ant is not able to distinguish the larger one among them.

**Figure 4:**
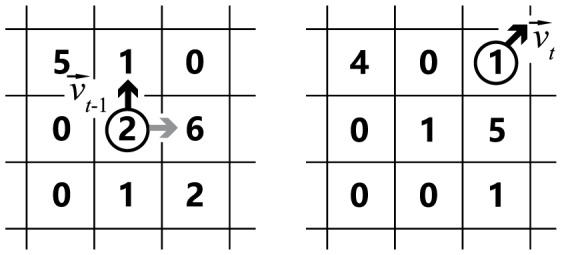
Schematic diagram demonstrating the rules of the alarm pheromone model. The left and the right panels show the updating rules in the model from time step 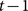 to 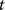, respectively. The circle denotes one ant, and the black arrows denote its current velocity vector. The numbers on the lattice denote the amount of pheromone, and the gray arrow pointing to 6 denotes the vector from the ant to the lattice where the concentration of pheromone is largest within its detection range. It should be noticed that the pheromone amount value in the simulation is not real world pheromone amount value. In our simulation, the amount of pheromone an ant puts on lattice and the amount of pheromone evaporates each time step are both less than 1. For simplicity, we use integers here.

The rules are described below, and a schematic diagram is presented in Fig. 4. We construct a two-dimensional square lattice chamber of 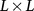.

1. Initially, each ant occupies one randomly chosen locus on the square lattice with a direction vector randomly chosen. (The detection range of an ant is its nearest 8 loci.)
2. At each time step, each ant leaves a constant amount of pheromone 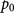 on the locus it resides to add up with the previously left pheromone by itself or others. The pheromone on each locus evaporates, to take the simplest form, a constant amount of 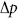 at each time step until it reaches zero.
3. When an ant detects one of the two exits, this ant escapes from the chamber immediately. When an ant meets the boundary, it reflects. Otherwise, the position of each ant updates according to the following equations:
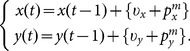
(4)

Here, 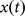 and 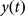 denote the 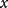 and 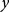 component of the position vector at time step 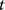, respectively. 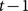 denotes the previous time step from time step 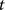. 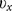 and 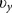 denote the 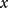 and 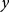 component of the velocity vector, respectively, which can only take the values of 1, 0 and −1. In addition, 0 cannot be assigned for both 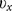 and 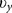 simultaneously. 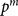 denotes the vector pointing to the detected maximum amount of pheromone from the ant, and 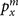 and 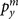 are the 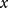 and 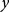 components. The bracket {} is defined as the formula below to ensure each ant moves exactly one lattice in every time step.
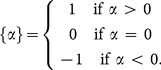
(5)


Moreover, if the detected amount of pheromone is larger than the given threshold 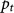, it is treated as 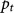. If more than one position where the pheromone amount is the same or larger than 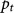, an ant selects a position randomly.

In the simulation, the following set of parameters is used: 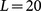, 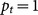, 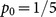 and 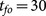 seconds. We set 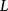 always to be 20, so that it corresponds to the ratio between the size of the cell (8.0 cm) and the average body length (0.39 cm) of the ants we used, which was determined by video measurement from our experiment. That is to say, one ant occupies one lattice exactly. Taking into account that the absolute values of 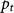 and 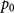 are meaningless, and only the relative values make sense, we set the threshold 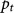 always to be 1 as a reference value. To compare the fade-out time of the pheromone in the simulation with that in the real world, we introduce 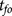 to denote the fade-out time from 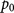 to 0. As the ants average speed is measured to be 0.94 cm/s, the relationship among 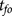, 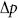 and 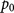 can be deduced as below:
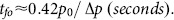
(6)


Experimental research shows that the fade-out time of the recruitment pheromone of the *Solenopsis invicta* is approximately 100 seconds [27]. For most species of ants, the components of alarm pheromones typically have a low molecular weight of 100–200 and consequently have a high volatility [20], [27], [28]. Thus, the fade-out time of alarm pheromone 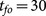 seconds is within the reasonable range.

**Figure 5:**
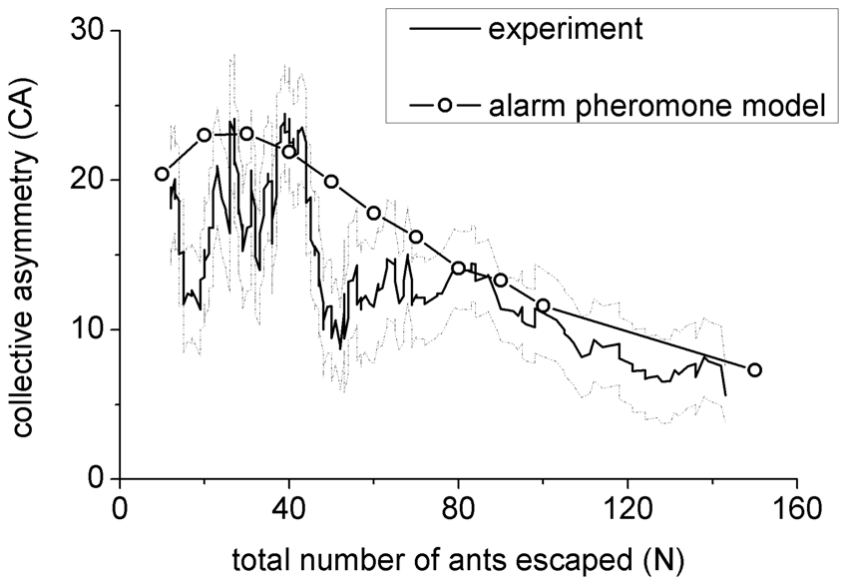
The comparison of the alarm pheromone model results and the experiment outcomes from Fig.2. The solid dots (simulation results) are averaged over 10000 runs with the parameters 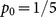 and 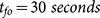. The model simulation agrees well with the experiment outcomes with the initial increase and then following decrease.

The simulation results compared with experimental results are shown in Fig. 5. The circles represent the simulation results averaged from 10000 runs. The simulation results suggest good agreement with the experiment outcomes, especially the first increasing then decreasing pattern with respect to the increase of ant number.

**Figure 6:**
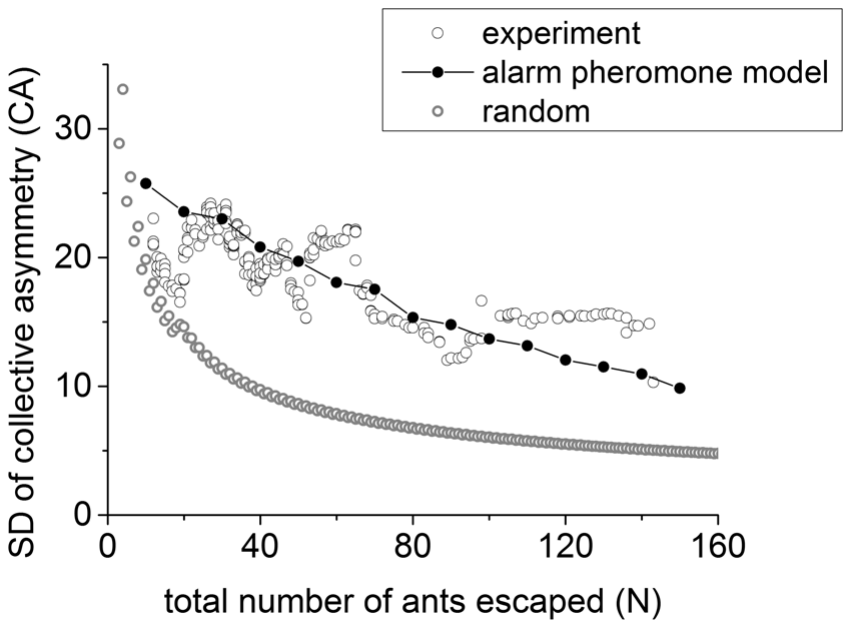
The standard deviation of 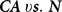. The simulation result (solid dots) produced by the alarm pheromone model agrees well with the experimental result (circle), and both differ from the random results (gray circle). The solid dots (simulation result) are averaged over 1000 runs.

In addition to the average collective asymmetry, we also investigated how the fluctuation of collective asymmetry is affected by total number of ants (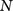). We use standard deviation (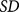) to denote the fluctuation. The experimental result displays a linear decrease of 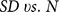. By using the same parameters as in Fig. 6, the simulation result agrees well with the experiment outcomes. Both the experimental results and simulation results, however, differ from random (Fig. 6). Just as we calculated the 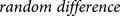 above, the 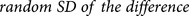 can be deduced (see *Materials and Methods*) supposing the possibility of escaping left and right for each ant is 0.5. When the 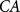 is large, approximately 40 ants (see Fig. 2), there is a greater difference of 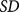 of 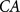 between the experimental results and random results (see Fig. 6).

#### Sensitivity Analysis

**Figure 7:**
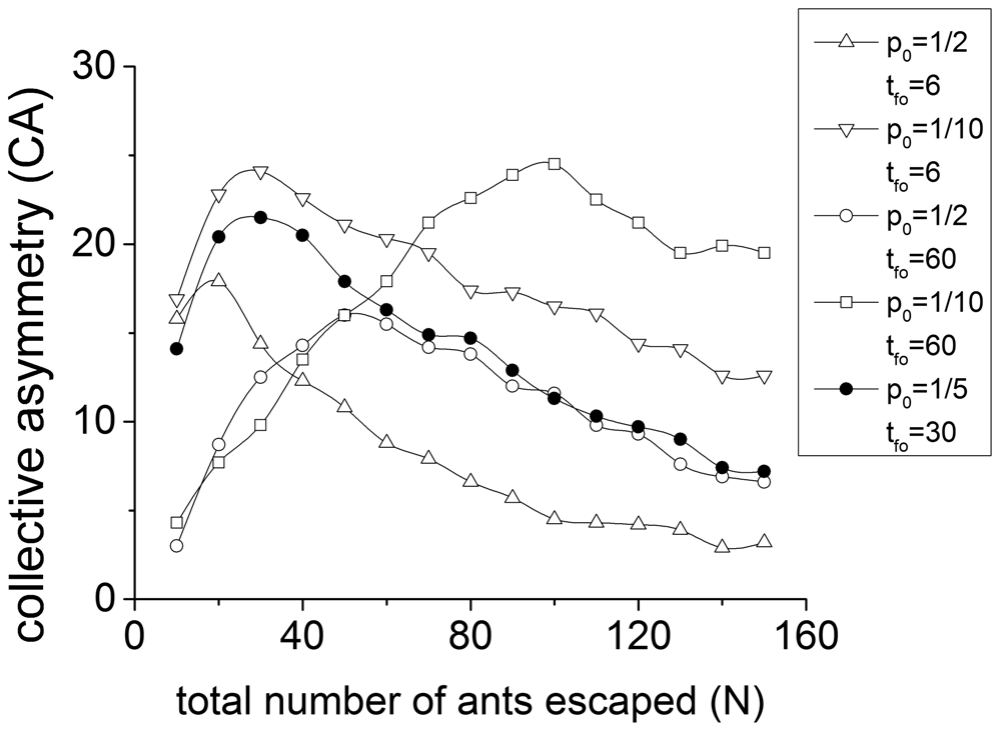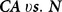 of alarm pheromone model. The solid dots denote the best-fit simulation result with the following parameters: 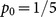 and 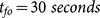. The others denote 4 combinations of different parameters when 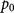 is assigned a value of 1/2 and 1/10, and 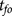 is assigned a value of 6 and 60 seconds. This demonstrates that the initial increasing and then decreasing pattern is robust. All the points are averaged over 1000 runs.

To check the robustness of the quantitative result of the alarm pheromone model, we change the values of the two parameters 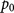 and 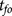. Each simulation is repeated 1000 times. The results are shown in Fig. 7. 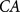 decreases with 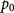 because when 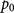 is large, the pheromone amount on the lattice can quickly reach 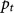, which is 1, such that the ants behave more similar to random particles. A small 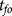 indicates high volatility. When the total number of ants is small, high volatility makes it difficult for the pheromone to accumulate, so the 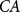 is small. However, when the total number of ants is large, the decrease of 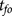 can keep the amount of pheromone as lower than 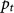, that is 1; therefore, the collective behavior in the ant group is more obvious, which leads to a higher 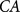. Generally speaking, the initial increasing and then decreasing pattern, which agrees with the experiment outcomes and quantitatively differs from the Vicsek-like model, is robust.

**Figure 8:**
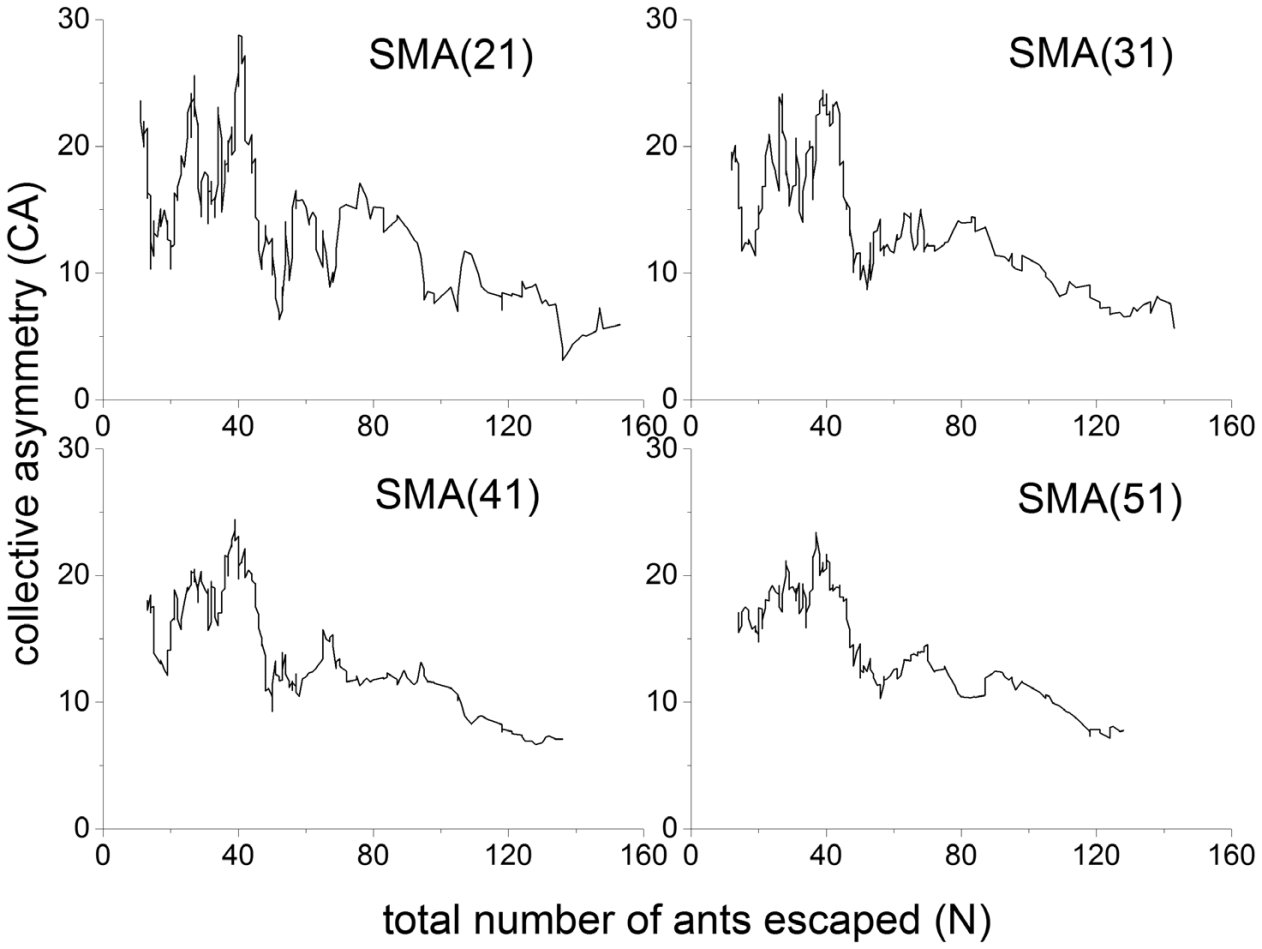
Moving average of the collective asymmetry (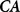) vs. the total number of escaped ants (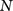) when changing the period of the moving average to be 21, 31, 41 and 51. These data indicate that the initial increasing and then decreasing pattern in the experiment does not depend on parameter values.

We also demonstrate the robustness of the 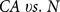 by changing the period of the moving average as shown in Fig. 8. As demonstrated, the initial increasing and then decreasing pattern in the experiment does not depend on the parameter values.

## Discussion

The experiments in this work demonstrate that there is an initial increase and subsequent decrease of the degree of collective asymmetry with the increase of the total number of escaped ants. To the best of our knowledge, this is the first systematic work with both experiments and modeling to study symmetry breaking relative to density. This paper provides results quite different from previous work reporting density has no significant impact [11] on symmetry breaking and the ever-increasing collective asymmetry in models with the alignment rule. The redefined collective asymmetry is measured with a deduction of the random-induced symmetry breaking from previous measures [11]. Based on the biological facts that ants use pheromones to communicate rather than seeing how other individuals move, we propose a simple yet effective alarm pheromone model. The model provides results that agree with the experimental outcomes.

An intuitive view of the alarm pheromone model is that the ant movement direction is affected by the accumulated pheromone. This enlarges the asymmetry of directions induced by random fluctuations and eventually leads to the asymmetry of escaped number at the macro level. The pheromone-mediated interaction is information sharing about the escape routes. The interaction occurs locally within a constant detection range, so the density comes to play an important role. When the volatility is high, at a very low density, the pheromone-mediated interaction among the ants can be too weak to produce asymmetry, and when the density increases, the asymmetry may increase accordingly. However, because of the limitations of biological perception, which is defined as the threshold to perceive different amounts of pheromone, too high of ant density leads to the deposition of so much pheromone that it undermines the distinguish ability and results in the decline of 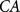 monotonously.

This research reveals that ants deposit and respond to alarm pheromone, and the accumulation of this biased information sharing leads to symmetry breaking, which suggests true fundamental rules of collective escaping behavior in ants.

## Materials and Methods

### Experiment

Before the experiments, the field-collected red imported fire ants (*Solenopsis invicta* Buren) from a single nest in South China Agricultural University located in Guangzhou city were fed in the laboratory for a few weeks, and the experiments were performed in the period of June 4–24, 2011. The temperature in laboratory is consistently approximately 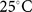. A group of ants were picked up with tweezers and immediately put into a small acquisition bottle, on the internal surface of which Fluon had been brushed and air dried, so that the ants were not able to climb up and could be transferred easily. A few seconds later, the ants in the bottle were introduced into the center of a circular cell that was 8 cm in diameter and 0.5 cm in height with two 1 cm wide exits symmetrically located left and right, which were initially blocked. The cell was rested on several layers, which were a piece of clipped circular filtering paper, a piece of thin plastic paper, and a piece of clipped A4 paper, from top to bottom. The cell was covered by a plastic plate immediately after the ants were introduced. The plastic cover was 0.3 cm in thickness with a hole of 0.3 cm diameter situated in the center. Then, a dose of 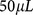 of an insect-repelling liquid (citronella, Labiofam, Cuba) was rapidly injected into the cell through the hole. The two exits were then opened synchronously so that the ants were able to escape. The time from picking up the ants to opening the exits was typically approximately 30 seconds. The whole setup rested on a horizontal plastic box without a cover that was 50 cm in length, 40 cm in width and 5 cm in height, which was divided into two parts symmetrically by plastic walls such that two isolated spaces were formed left and right for retaining and counting the escaped ants. Two desk lamps were placed beside the box symmetrically to maintain a close to uniform light intensity and thus prevent a possible moving direction preference of the ants being induced by uneven light intensity. The whole process was recorded until the end of the escape activity using a video camera situated above the cell.

There were 291 repetitions in total. In each repetition, a new group of ants and new layers under the cell (including the filtering paper, plastic paper and A4 paper) were used to avoid residual liquid and possible pheromone residue on the equipment.

It was impossible to precisely control the total number of ants in each experiment. The reason is twofold. First, to keep the ants in a high-panic situation, the ants have to be introduced into the cell quickly enough at the cost of impreciseness of the ant number. Secondly, some ants died or gradually lost mobility because they contacted the repellent oil before the oil had been blotted by the filtering paper. The average proportion of the remaining ants at the end of each experiment to the total number of ants at the beginning of each experiment is 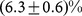. The total number of ants escaped in the experiments ranged from 6 to 269.

### Definition of symmetry breaking

Considering an 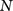 particle random SPP model, for an unbiased movement, the possibility of escaping left and right for each particle should be equal, that is 1/2. After some simple statistical derivations, we obtain the random difference produced from an 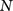 particle random SPP model as
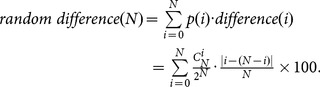
(7)where 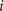 denotes the total number of ants escaping left. 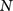 denotes the total number of escaping ants. 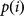 and 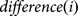 denote the possibility and difference that the total of ants escaping left is 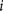, respectively.

### Definition of random SD of difference

Just as we calculated the 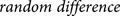 above, the random 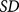 of the difference can be deduced supposing the possibility of escaping left and right for each ant is 0.5.
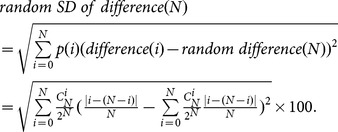
(8)


## Supporting Information

S1 Data (XLSX)
